# Supplementary material for: Investigating the Biocontrol Potential of the Natural Microbiota of the Apple Blossom
Source: Microorganisms. 2022 Dec 15;10(12):2480. doi: 10.3390/microorganisms10122480 (PMC9784478; doi:10.3390/microorganisms10122480)
Supplement: Supplementary file 1 [file microorganisms-10-02480-s001.zip › microorganisms-2076844-Figures.pdf]

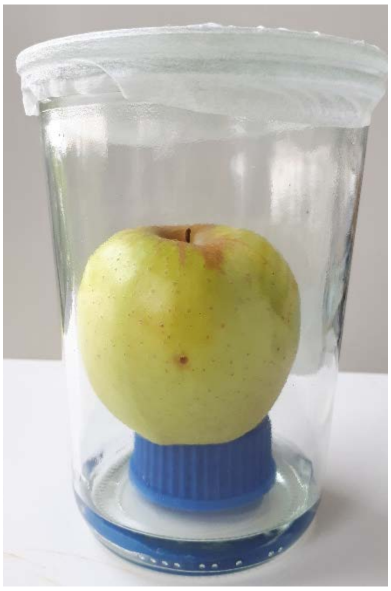

Figure S1: Apple model for testing activity against *E. amylovora*. Injected apple before incubation.

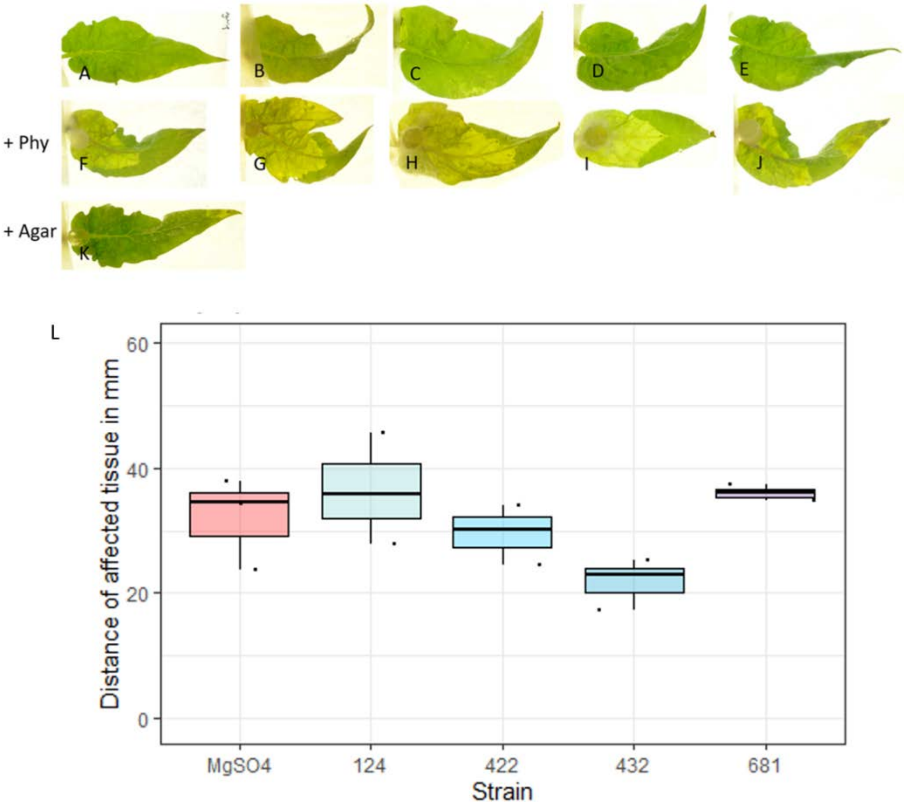

Figure S2: Tested strains did not inhibit *P. infestans*. A-K) Tomato leaves sprayed with $MgSO_4$ solution (A,F,K), #124 (B,G), #422 (C,H) , #432 (D,I), #681 (E,J) and inoculated with an agar plug containing *P. infestans* (+Phy) or only agar (+ Agar). The distance of water soaking progression across the leaf was measured and plotted.

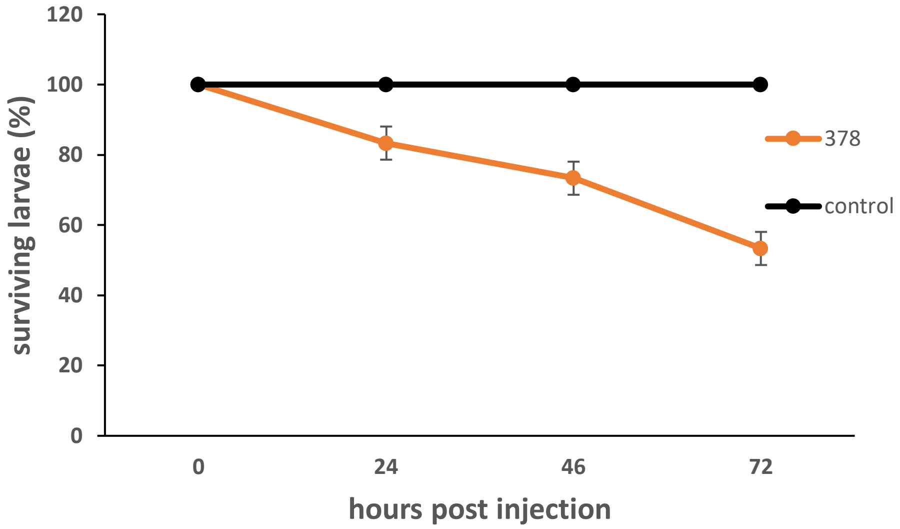

Figure S3: Survival curve for *G. mellonella* larvae infected with *P. agglomerans* #378. Larvae were injected with approximately 5 × 10^4 bacteria and incubated at 30°C in the dark. Live and dead larvae were counted at 24, 48 and 72 h p.i. Curve represents the mean of three separate experiments. Error bars represent the standard deviation of the data.
